# Overweight and Obesity in Schoolchildren: Hierarchical Analysis of Associated Demographic, Behavioral, and Biological Factors

**DOI:** 10.1155/2018/6128034

**Published:** 2018-09-05

**Authors:** Cézane P. Reuter, Elza D. de Mello, Priscila T. da Silva, Tássia S. Borges, Elisa I. Klinger, Silvia I. R. Franke, Andréia R. de M. Valim

**Affiliations:** ^1^Physical Education and Health Department, Postgraduate Program in Health Promotion, University of Santa Cruz do Sul (UNISC), 96.815-900 Santa Cruz do Sul, RS, Brazil; ^2^Postgraduate Program in Child & Adolescent Health, Federal University of Rio Grande do Sul (UFRGS), 90.035-003 Porto Alegre, RS, Brazil; ^3^Postgraduate Program in Health Promotion, University of Santa Cruz do Sul (UNISC), 96.815-900 Santa Cruz do Sul, RS, Brazil; ^4^School of Dentistry, Lutheran University Center of Palmas (CEULP-ULBRA), 77.019-900 Palmas, TO, Brazil; ^5^Biology and Pharmacy Department, Postgraduate Program in Health Promotion, University of Santa Cruz do Sul (UNISC), 96.815-900 Santa Cruz do Sul, RS, Brazil

## Abstract

Studies focused on the mechanisms involved in the development of obesity in children and adolescents have reported associations between this condition and birth weight, sedentary lifestyle, and hereditary conditions. However, few studies have simultaneously evaluated these factors. This cross-sectional study aims to identify demographic, behavioral, and biological factors associated with overweight/obesity in children and adolescents. 381 schoolchildren aged seven to 17 years were included in the study to evaluate the associations between overweight/obesity and biological factors (including family history of obesity, birth weight, and the fat mass and obesity-associated (FTO) rs9939609 polymorphism), demographic variables (including gender and age), and behavioral variables (including physical activity and/or sports participation). The results of this study showed that there was a lower prevalence of obesity in schoolchildren aged 11–17 years (PR: 0.89; *p*=0.004). Obesity was more prevalent in children whose father (PR: 1.24; *p* < 0.001) and maternal grandmother (PR: 1.16; *p*=0.019) were obese. Higher prevalence rates of obesity were also identified in schoolchildren who were overweight at birth (PR: 1.18; *p*=0.002) and carriers of the obesity risk genotype (PR: 1.13; *p*=0.016). Biological factors, such as family history of obesity, overweight at birth, and the presence of the fat mass and obesity-associated rs9939609 polymorphism were associated with the prevalence of obesity in children and adolescents.

## 1. Introduction

The mechanisms underlying the development of obesity in children and adolescents have been the topic of intense research, and studies are perpetually indicating different sets of factors that may be associated with these conditions [1]. Recent studies have suggested a relationship between birth weight, obesity, and sedentary lifestyle [1, 2]. In addition, it has been suggested that birth weight may be explained by hereditary factors and associated with maternal body mass index (BMI) and paternal eating behaviors. These factors constitute important health aspects that may lead to an increased risk of developing an altered metabolic profile [3, 4].

In this way, the use of birth weight as a health determinant has been increasingly supported because it allows for a set of data related to health outcomes, mainly in childhood and adolescence, to be predicted [5]. Previous studies have indicated that high birth weight infants are 2.5 times more likely to become obese children and adolescents [6]. In addition, adolescents with either high or low birth weight are at increased risk of developing insulin resistance, hyperleptinemia, adiposity, and metabolic disorders [7].

Similarly, the results of the Healthy Lifestyle in Europe by Nutrition in Adolescence (HELENA) study, which included 3,528 children from European cities, suggested that children in the 9-year-old age group with a low physical fitness level were at elevated risk of developing cardiovascular risk factors such as obesity and overweight [8].

Epidemiological studies of other mechanisms involved in genetic inheritance have noted that the FTO gene polymorphism may be associated with obesity, and data suggest that children and adolescents with FTO gene polymorphism rs9939609, especially with the AA and AT risk allele, may be more likely to become obese [9, 10]. Therefore, exploring and ascertaining associations between the behaviors of children and parents may be an efficient manner in which to reduce the prevalence of diseases associated with the development of obesity in this population. Thus, the present study aimed to identify demographic, behavioral, and biological factors associated with overweight/obesity in children and adolescents.

## 2. Materials and Methods

This cross-sectional study included a sample of 381 schoolchildren aged seven to 17 years from nine schools in urban and rural areas of the city of Santa Cruz do Sul, Rio Grande do Sul State, southern Brazil. The study sample was of convenience, with a draw in each classroom for the election of the students participating. The study was approved by the Committee of Ethics in Research with Human Beings of the University of Santa Cruz do Sul (UNISC) under protocol number 2959/11, in accordance to Declaration of Helsinki. All parents or guardians signed the free and informed consent form.

The G^*∗*^Power 3.1 program (Heinrich-Heine-Universität Düsseldorf, Germany) was used to calculated the sample size needed for a Poisson regression (presence versus absence of obesity as a dependent variable). Using the calculation parameters recommended by Faul et al. [11], we determined that a minimum sample of 372 students would be required to detect an effect size of 0.30 at a power (1-*β*) of 0.80 and significance level of *α* = 0.05.

We used the BMI to evaluate the presence of overweight/obesity, which was calculated using the following formula: BMI = weight/(stature)2. The values obtained using this formula were classified according to the cutoff points established by the World Health Organization [12] Subsequently, we classified subjects into the following two categories: low weight/normal weight and overweight/obesity. Demographic variables (sex and age) were self-reported by the schoolchildren and later validated via data obtained from the school. Subsequently, age was classified into the following two categories: child (up to 12 years) and adolescent (up to 18 years), as established by the Child and Adolescent Statute [13]. This choice of age group assumed to perform a comparison between ages and observe what would be more related to BMI. In this way, knowing the influences of age, we may act on prevention and health promotion programs, focusing mainly on major influencers. Maternal education was self-reported by parents. The evaluated behavioral variable was participation in physical/sports activities, as reported by the school. A questionnaire adapted from that of Barros and Nahas [14] was used, with categories comprising “never,” “sometimes/almost always,” and “always.”

To identify potentially associated biological factors, family history of obesity (father, mother, siblings, and maternal and paternal grandparents), and birth weight were evaluated via a questionnaire completed by the parents of the students. Birth weight, obtained in grams, was later classified as “underweight/insufficient weight,” “adequate weight,” and “overweight,” according to the Puffer and Serrano classification [15].

In addition, the presence of the rs9939609 polymorphism in the fat mass and obesity-associated gene (FTO) gene was evaluated. The rs9939609 polymorphism was selected because it has been found to have a higher allelic frequency in the target population of the study, which was predominantly Caucasian [16, 17]. Deoxyribonucleic acid (DNA) was extracted from whole blood samples containing ethylenediaminetetraacetic acid (EDTA) using the Qiagen Kit (QIAamp DNA Blood Mini Kit, Qiagen, Germany) and subsequently quantitated using the Qubit® 2.0 fluorometer (Invitrogen, Carlsbad, CA, USA). Polymorphism genotyping was carried out via real-time polymerase chain reactions (PCRs), which were performed in a 96-well format in a total of 5 *μ*l reaction volume using 10 ng of genomic DNA and VIC®/FAM™ dye tagged TaqMan probes (Applied Biosystems, Foster City, CA, USA) in StepOnePlus (Applied Biosystems, Foster City, CA, USA). The number of cycles and the temperature used for the PCRs were determined according to the manufacturer's recommendations. All genotyping was performed in duplicate, showing an accuracy of 100%. The Hardy–Weinberg equilibrium was tested (*p* > 0.05).

Data analysis was performed using SPSS 23.0 for Windows software (IBM, USA). First, descriptive analyses of the exposure and outcome variables were performed, and absolute and relative values were calculated. In the multivariate analysis, a Poisson regression was used, which has demonstrated robust variance (prevalence ratio (PR) with 95% confidence interval). A hierarchical model was used for the analysis, and the variables were categorized into the following groups, which are organized from the most distal level (furthest from the outcome) and to the most proximal level (closest to the outcome): (a) sociodemographic (sex, age, and maternal education); (b) behavioral (physical/sports activity); and (c) biological (familial obesity, birth weight, and FTO rs9939609). For the final models, the exposures were entered into the model by level and selected via backward stepwise selection (variables with *p* < 0.20 were included in the multivariable analysis). Variables with *p* < 0.05 in the final model were considered statistically significant.

## 3. Results and Discussion

The descriptive characteristics of the sample are presented in Table 1. Of the 381 students evaluated, 50.4% were female, and the prevalence of overweight/obesity was 35.7%. The obesity risk allele (A) was present in 57.5% of the students.

Table 2 presents the associations between the evaluated demographic, behavioral, and biological factors and overweight/obesity in schoolchildren. We observed that overweight/obesity was less prevalent (PR: 0.89; *p* < 0.001) in adolescents than children. Several biological factors, such as having an obese father (PR: 1.24; *p* < 0.001) and maternal grandmother (PR: 1.16; *p*=0.01), being overweight at birth (PR: 1.18; *p* < 0.001), and having the obesity risk genotype (AA) rs9939609 polymorphism (PR: 1.13; *p*=0.01) were associated with greater overweight/obesity prevalence rates in schoolchildren.

## 4. Discussion

Our findings describe the associations observed between demographic and biological factors and overweight/obesity. In this study, a lower prevalence of obesity was identified in adolescents (PR: 0.89; *p*=0.004). Although recent studies have also reported there to be high prevalence rates of obesity in both children and adolescents [18, 19], similar findings were found in a study recently conducted by Jagadesan et al., which reported there to be a lower prevalence of obesity in adolescents, suggesting that the prevalence of overweight and obesity did not increase with age in the evaluated population, except for a small increase observed between 10 and 12 years of age [20]. However, it has been reported that obese children and adolescents may be more likely to remain obese during adulthood [21]. In addition, current evidence points to a 47% increase in the prevalence of obesity in children between 1980 and 2013 [22]. These data demonstrate the need to develop and direct more complex actions and research to prevent obesity in early childhood. Although in our sample the highest percentage of examined have been teenagers, these showed a smaller percentage of obesity. This result may have been influenced by the wide age range of subjects of this study, which involve children and adolescents; however, statistical analysis was performed in order to avoid/reduce effects of possible/any confounding variable.

Our results showed a higher prevalence of the FTO genotype in students with the risk allele (A) for obesity (57.5%). The results of a study previously carried out in Santa Cruz do Sul suggested that a high prevalence of obesity has been maintained in children and adolescents, and of students with the AA genotype, 57.4% were overweight/obese [16]. Meng and coworkers noted that polymorphisms in the FTO gene (rs62048402 and rs9939609) had significant effects on BMI [23]. Thus, these data emphasize the contribution of genetic factors in the etiology of obesity [24–26], which has been further supported by findings indicating that the heritability of BMI can vary from 30 to 70% [25].

In addition, our findings showed that students with the AA genotype had a 13% greater prevalence of obesity. Therefore, the results of this study support the existence of associations between the variants of the FTO gene and obesity. Similar estimates were identified for variants of the FTO gene in a study of 15,580 Chinese children aged seven to 18 years from the cities of Beijing, Tianjin, Chongqing, Hangzhou, Shanghai, and Nanning. The study indicated that participants with allele A (AA and AT) had a higher prevalence of obesity (OR: 1.47 and OR: 3.32, respectively). In addition, obesity-related metabolic traits, such as increased BMI; waist circumference; hip circumference; and triglyceride, low-density lipoprotein cholesterol (LDL-c), and glucose and decreased high-density lipoprotein cholesterol (HDL-c) levels, were more frequently identified in participants with the AT or AA genotype than participants with the TT genotype [10]. However, this result needs to be further investigated.

The results of the study suggest there to be an association between obesity/obesity in children and adolescents and familial history of diabetes, with schoolchildren who had an obese father and obese maternal grandmother having 24% and 16% greater prevalence rates of overweight/obesity, respectively. Similar findings were previously observed in Tuscany, Italy, where the results of a cross-sectional study of 1,751 children between eight and nine years of age suggested that the prevalence of obesity among children with mothers of normal weight was only 1.4%, whereas the prevalence of obesity among children with obese mothers was 30.3%. Similarly, the prevalence of obesity among children with normal weight fathers was 4%, and the prevalence of obesity among children with obese fathers was 23.9% [27]. In the cities of Catania and Sicily, Italy, the results of a cross-sectional study involving 1,521 children and adolescents suggested that excess birth weight (≥4.0 kg; OR: 1.83; *p* < 0.05), overweight or obese mother (OR: 2.33, *p* < 0.0001), obese father (OR: 1.68; *p* < 0.001), and low/medium parental education level (OR: 1.72; *p* < 0.005) were associated with increased odds of youth obesity development. In addition, breastfeeding was identified as a protective factor (OR: 0.64; *p* < 0.0005) [28].

Our results also indicated that schoolchildren who were overweight at birth had an 18% higher prevalence of overweight/obesity than schoolchildren who had a normal weight at birth. These findings provide additional evidence supporting the presence of a relationship between birth weight and overweight/obesity. A study carried out with 470 schoolchildren in Salvador, Bahia, showed that high birth weight was associated with high BMI and complications such as obesity, changes in lipid profile, and cardiovascular diseases [2]. Thus, the authors have previously argued that both low birth weight and high birth weight may be associated with elevated health risks among schoolchildren [29]. However, underweight/insufficient weight at birth was not associated with overweight/obesity in our study.

A study conducted in Michigan, USA, with two to five-year-old children and their mothers showed similar findings, indicating that newborn children with a BMI above the 90th percentile had a 2.5-fold higher prevalence of obesity [7]. In adolescents, high birth weight has been reported to be associated with cardiovascular risk factors, such as obesity and high blood pressure [2]. Thus, the findings of the present study demonstrated that birth weight was associated with obesity in childhood and adolescence, which may allow for the early identification of metabolic risk and birth weight variations that could trigger cardiometabolic complications during later stages of life. However, it is important to note that birth weight variations should not be considered as a cause of health compromises in isolation, given the innumerable opportunities for changes in lifestyle.

Thus, the datasets used in our study helped provide increased understanding regarding the complexity of the etiology of obesity in children and adolescents. In general, our data provided further evidence suggesting that the control and management of obesity requires a holistic and interdisciplinary view that respects the individualities of each child and that offers conditions within which effective changes can be made, including hereditary factors, biological, and lifestyle factors. However, it should be noted that the study had limitations. Because it was a cross-sectional study, it was not possible to establish causality. In addition, the birth weight, physical activity participation, and BMI variables were self-reported by parents, which may have biased the observed associations. Also, the weeks of gestation were not evaluated, being considered an important vies of the study. However, the study utilized a very broad approach in assessing conditions related to the health of children and adolescents and identifying factors relevant to the health of Brazilian children. In addition, the results of the study suggest that new analyses involving the collection of physical activity data through objective evaluations should be performed to better understand this association.

## 5. Conclusion

Biological factors, such as family history of obesity, including paternal and matrilineal obesity, overweight at birth, and the presence of the fat mass and obesity-associated rs9939609 polymorphism, were associated with the presence of overweight/obesity in children and adolescents.

## Figures and Tables

**Table 1:** Sample characteristics (*n*=381).

Variables	*n* (%)
Sex	
Male	189 (49.6)
Female	192 (50.4)
Age group	
Children	134 (35.2)
Adolescents	247 (64.8)
BMI	
Low weight/normal weight	245 (64.3)
Overweight/obesity	136 (35.7)
Birth weight	
Underweight/insufficient weight	117 (30.7)
Adequate weight	229 (60.1)
Overweight	35 (9.2)
FTO rs9939609	
TT	162 (42.5)
AT/AA	219 (57.5)
Maternal education	
≤4 years	121 (32.5)
5 to 11 years	204 (54.8)
≥12 years	47 (12.7)

BMI: body mass index; FTO: fat mass and obesity-associated gene.

**Table 2:** Crude and adjusted analyses of the prevalence of obesity and associated factors.

Variables		Crude model (CI 95%)	*p*	Adjusted model (CI 95%)	*p*
**Level 1**: sociodemographic					
Sex					
Male		1.00		1.00	
Female		0.95 (0.89–1.02)	0.23	0.95 (0.89–1.02)	0.20
Age group					
Children		1.00		1.00	
Adolescents		0.88 (0.82–0.95)	0.00	0.89 (0.83–0.96)	0.00
Maternal education					
≤4 years		0.93 (0.83–1.05)	0.29	0.95 (0.84–1.07)	0.44
5 to 11 years		1.00 (0.89–1.11)	0.99	1.00 (0.89–1.11)	0.98
≥12 years		1.00		1.00	

**Level 2**: behavioral					
Physical/sports activity					
Never		1.00 (0.90–1.11)	0.970	1.00 (0.90–1.11)	0.94
Sometimes/almost always		1.03 (0.95–1.12)	0.364	1.04 (0.96–1.12)	0.33
Always		1.00		1.00	

**Level 3**: biological					
Obese father					
Yes		1.25 (1.09–1.44)	0.001	1.24 (1.08–1.41)	0.00
No		1.00		1.00	
Obese mother					
Yes		1.06 (0.93–1.20)	0.366	0.92 (0.81–1.05)	0.25
No		1.00		1.00	
Obese siblings					
Yes		0.92 (0.65–1.29)	0.634	0.93 (0.62–1.38)	0.72
No		1.00		1.00	
Obese maternal grandmother					
Yes		1.15 (1.02–1.31)	0.021	1.16 (1.02–1.32)	0.01
No		1.00		1.00	
Obese maternal grandfather					
Yes		1.13 (0.95–1.·36)	0.156	1.02 (0.83–1.26)	0.80
No		1.00		1.00	
Obese paternal grandmother					
Yes		1.05 (0.90–1.23)	0.484	0.95 (0.81–1.12)	0.58
No		1.00		1.00	
Obese paternal grandfather					
Yes		1.11 (0.97–1.43)	0.085	1.00 (0.81–1.23)	0.99
No		1.00		1.00	
Birth weight					
Underweight/insufficient weight		0.94 (0.87–1.02)	0.149	0.94 (0.87–1.02)	0.18
Overweight		1.17 (1.05–1.31)	0.004	1.18 (1.06–1.31)	0.00
Adequate weight		1.00		1.00	
FTO rs9939609				
TT		1.00		1.00	
AT		0.96 (0.89–1.04)	0.393	0.95 (0.89–1.03)	0.26
AA^*∗*^		1.17 (1.06–1.30)	0.002	1.13 (1.02–1.25)	0.01

^*∗*^Obesity risk genotype; CI: confidence interval; FTO: fat mass and obesity-associated gene; Poisson regression analysis with presence of overweight/obesity evaluated as outcome variable.

## Data Availability

Data in our manuscript are not available for sharing as they are owned by our university.
